# Kindling in Alcohol Withdrawal

**Published:** 1998

**Authors:** Howard C. Becker

**Affiliations:** Howard C. Becker, Ph.D., is an associate professor in the departments of psychiatry and behavioral sciences and physiology, Medical University of South Carolina, and an associate research career scientist at the Veterans Affairs Medical Center, Charleston, South Carolina

**Keywords:** AOD withdrawal syndrome, AODR (alcohol and other drug related) seizure, symptom, disease severity, neurotransmission, neurotransmitter receptors, cell electrophysiology, sensory stimuli, biochemical mechanism, AOD abstinence, AODD (alcohol and other drug dependence) relapse, brain damage, cognitive process, detoxification, treatment, animal model, clinical study, literature review

## Abstract

In many alcoholics, the severity of withdrawal symptoms increases after repeated withdrawal episodes. This exacerbation may be attributable to a kindling process. Kindling is a phenomenon in which a weak electrical or chemical stimulus, which initially causes no overt behavioral responses, results in the appearance of behavioral effects, such as seizures, when it is administered repeatedly. Both clinical and experimental evidence support the existence of a kindling mechanism during alcohol withdrawal. Withdrawal symptoms, such as seizures, result from neurochemical imbalances in the brain of alcoholics who suddenly reduce or cease alcohol consumption. These imbalances may be exacerbated after repeated withdrawal experiences. The existence of kindling during withdrawal suggests that even patients experiencing mild withdrawal should be treated aggressively to prevent the increase in severity of subsequent withdrawal episodes. Kindling also may contribute to a patient’s relapse risk and to alcohol-related brain damage and cognitive impairment.

Alcohol dependence and alcohol abuse frequently involve drinking patterns in which bouts of heavy drinking (i.e., binge drinking) are interspersed with periods of abstinence. During the binge-drinking episode, the body, particularly the brain, adapts to the presence of alcohol by compensating for alcohol’s effect on the central nervous system (CNS). Alcohol has an overall suppressing effect on CNS activity. Accordingly, the adaptation process involves several mechanisms to increase the excitability of nerve cells (i.e., neurons) in the brain—that is, their ability to become activated in response to signals from other neurons. When the alcohol is eliminated from the body during abstinence, this compensatory activation of the CNS remains in effect for several more days, resulting in excessive excitability of the CNS (i.e., hyperexcitability). This hyperexcitability manifests itself as alcohol withdrawal (AW), with symptoms ranging from tremors and agitation to seizures and delirium tremens. As a result of the bingeing-abstaining consumption pattern, many alcoholics experience numerous withdrawal episodes during the course of their illness (Hillbom 1990).

The severity of AW symptoms can differ widely among alcoholics and even among different withdrawal episodes in the same person. Both the amount of alcohol consumed and the duration of intoxication just before cessation of drinking are important determinants of the severity of a withdrawal reaction. In addition, a history of withdrawal episodes appears to be a critical factor in the intensity of withdrawal symptoms. Accordingly, some researchers have suggested that repeated AW may sensitize a person to subsequent withdrawal episodes. This hypothesis implies that the severity of withdrawal-related symptoms may increase in a cumulative fashion, with more severe symptoms becoming evident after years of alcohol abuse and numerous periods of abstinence.

The mechanisms underlying the exacerbation of withdrawal symptoms following repeated withdrawal episodes are currently unknown. One hypothesis proposes that the phenomenon may result from a “priming” effect, in which each consecutive episode of alcohol exposure evokes stronger compensatory (i.e., withdrawal) responses. In contrast, Ballenger and Post (1978) have hypothesized that the progressive exacerbation of AW is the manifestation of a “kindling” mechanism, which has been observed in other neurological conditions. According to this model, it is the repeated experience of AW, rather than repeated alcohol exposure, that underlies the progressive intensification of symptoms. Although more evidence supports such a kindling mechanism, a priming effect cannot be ruled out. In fact, the two mechanisms are not mutually exclusive, and both may operate in the final expression of the phenomenon (Becker and Littleton 1996).

Over the past two decades, numerous studies have investigated the phenomenon of withdrawal sensitization (Becker and Littleton 1996). This article provides an overview of clinical and experimental research findings supporting the kindling hypothesis of AW. The article also explores the mechanisms underlying kindling and addresses implications of kindling related to AW treatment strategies and to long-term consequences of alcoholism.

## Kindling as a Mechanism of Neural Sensitization

The term “kindling” was first introduced by Goddard and colleagues (1969) to describe a phenomenon observed after repeated weak electrical stimulation of discrete brain regions using electrodes implanted into the brain. The researchers found that the electrical stimuli initially produced no overt behavioral effects, such as seizures (i.e., the stimuli were subconvulsive). After repeated periodic application, however, the same subconvulsive stimuli induced the development of full motor seizures, suggesting that the brain had become sensitized to the stimulation. Additional studies found that sensitization could occur regardless of whether the stimulus was electrical or chemical in nature (e.g., a convulsant drug administered directly into the brain or into other parts of the body) (Gilbert 1992; Post et al. 1988). For kindling to occur, however, it is critical that the subconvulsive stimulus be administered in a repeated, intermittent fashion. This requirement is a unique characteristic of kindling.

The kindling process is associated with increased neuronal excitability, whose time course corresponds to that of the progressive intensification of behavioral and convulsive responses. Furthermore, once the enhanced brain excitability and susceptibility to convulsions has been established, it can last for several months. This durability of the kindling phenomenon most likely reflects long-term changes in neuronal circuitry and function (McNamara and Wada 1997).

When applying the kindling hypothesis to AW, researchers have postulated that each withdrawal-induced episode of CNS hyperexcitability may serve as a stimulus that supports a kindling process (Ballenger and Post 1978). Accordingly, although binge drinking may not initially result in serious, or even noticeable, withdrawal symptoms, repeated episodes of this pattern of alcohol intoxication followed by abstinence and withdrawal may lead to a worsening of future withdrawal-related symptoms. Thus, a kindling process may underlie the commonly observed progression of withdrawal symptoms from relatively mild responses (e.g., irritability and tremors) characteristic of initial withdrawal episodes to more severe symptoms (e.g., seizures and delirium tremens) associated with subsequent withdrawal episodes (Ballenger and Post 1978).

## Kindling in Alcohol Withdrawal

### Clinical Evidence

A growing body of clinical evidence supports the kindling hypothesis for AW. Several clinical studies that retrospectively reviewed patient records revealed that hospitalized alcoholics who suffered a seizure during detoxification were more likely to have a history of numerous withdrawal episodes than were hospitalized alcoholics who did not suffer seizures. For example, Brown and colleagues (1988) found that 48 percent of inpatient alcoholics who had seizures during detoxification had experienced five or more previous withdrawal episodes, whereas only 12 percent of a control group of hospitalized alcoholics who experienced no seizures had such a history. More recent studies have corroborated these findings, demonstrating a positive correlation between the occurrence of seizures during withdrawal and a history of previous detoxifications (e.g., Lechtenberg and Worner 1991; Moak and Anton 1996).

An increased risk of seizures in alcoholics with a history of multiple withdrawal episodes is clinically significant, because patients experiencing AW-related seizures have a poorer prognosis and a higher mortality rate compared with people with seizures from unknown causes (Pieninkeroinen et al. 1992). Booth and Blow (1993) conducted a retrospective analysis of admission records for 6,818 male alcoholics treated at 172 U.S. Veterans Affairs Hospitals. Their study found that a history of detoxification was associated with a significantly increased likelihood of hospital readmission for alcoholism and alcohol-related problems. Moreover, previous withdrawal episodes were associated with more severe and medically complicated withdrawal, although the mortality from AW-induced seizures was not significantly influenced by the patient’s withdrawal history (Booth and Blow 1993; Pieninkeroinen et al. 1992). Collectively, these results support the notion that after multiple withdrawal episodes, patients become sensitized (possibly through a kindling mechanism) and subsequently experience more severe withdrawal symptoms.

### Experimental Evidence

The use of animal models has further validated the kindling concept of AW and substantiated many of the observations noted in clinical settings. For example, several animal studies have demonstrated increased neural hyper-excitability and exacerbation of withdrawal symptoms following repeated withdrawal experiences (Becker and Littleton 1996). This experimental support is particularly relevant, because numerous concurrent and intervening variables associated with alcoholism that may affect kindling (e.g., malnutrition and variable periods of alcohol exposure and abstinence) cannot be adequately controlled in studies in humans and may confound interpretation of results. Animal models, in contrast, frequently allow analysis of the kindling effect under conditions in which variables such as intensity (i.e., blood alcohol levels) and duration of alcohol exposure, duration of abstinence, general health, and nutritional status can be rigorously controlled.

Animal models to investigate kindling of withdrawal symptoms have used several different methods of chronic alcohol exposure, including alcohol administration through a tube directly into the stomach, feeding of an alcohol-containing liquid diet, and exposure to alcohol vapors (for a review, see Anton and Becker 1995). These studies found that withdrawal symptoms were more severe in animals with previous withdrawal experience than in animals that experienced first-time withdrawal. Moreover, the results suggested that some withdrawal symptoms (e.g., seizures) may be particularly susceptible to kindling.

Accordingly, many studies have focused on kindling of withdrawal seizures. For example, alcohol intoxication achieved by exposing mice to alcohol vapors for 16 hours in inhalation chambers resulted in a mild withdrawal response when the animals experienced a first withdrawal episode. After repeated intoxication-withdrawal experiences, however, the same alcohol exposure induced progressively more severe withdrawal-related convulsions (Becker 1994). Other studies also found that both the intensity and the duration of exacerbated withdrawal seizures increased with the number of previous withdrawal experiences.

The significance of repeated withdrawal also has been demonstrated in experiments that compared withdrawal responses in two groups of mice exposed to the same amount of alcohol but according to different exposure schedules (e.g., Becker and Hale 1993; Becker et al. 1997). In these experiments, one group of animals was continuously exposed to alcohol vapors for 48 hours, whereas the second group was exposed to alcohol for a total of 48 hours divided into three cycles of 16 hours of intoxication separated by 8 hours of abstinence. The analyses found that mice subjected to interrupted alcohol exposure exhibited more severe withdrawal seizures compared with the animals subjected to uninterrupted alcohol exposure. Moreover, the differences in withdrawal responses did not appear to be related to alterations in the rate of alcohol elimination from circulation following withdrawal. These results suggest that repeated AW, rather than the amount of alcohol exposure per se, may be critical for the kindling of AW symptoms.

Researchers have used animal models to investigate various additional issues related to kindling in AW with the following results:

- The exacerbated behavioral symptoms (e.g., convulsions) observed in animals with a history of repeated withdrawal episodes were accompanied by progressively greater changes in brain activity, as determined by electroencephalography (EEG) (Poldrugo and Snead 1984; Veatch and Gonzalez 1996; Walker and Zornetzer 1974). These changes appear as abnormal patterns of brain waves (i.e., spikes and sharp waves) in specific brain regions. More detailed analyses found that the EEG patterns in some brain regions may be particularly sensitive to the individual’s history of withdrawal episodes, whereas the EEG activity of other brain sites may be more responsive to total amount of alcohol exposure before withdrawal (Veatch and Gonzalez 1996). (For more information, see sidebar by Gonzalez, pp. 34–37.)
- Repeated AW influences the development of subsequent kindling not only in response to alcohol but also in response to electrical stimuli. Whether the susceptibility to electrical kindling increases or decreases after repeated withdrawal episodes depends on the specific brain regions being studied (McCown and Breese 1990; Veatch and Gonzalez 1997).
- Repeated withdrawal experiences result in changes in local metabolic activity (e.g., how the brain metabolizes the sugar glucose) in numerous brain regions (Clemmesen et al. 1988). These metabolic changes may reflect alterations in the electrical activity (i.e., EEG patterns) of those brain regions.
- Studies with mice, rats, and primates have shown that a history of withdrawal decreases the duration and extent of intoxication necessary to provoke a subsequent withdrawal response upon cessation of alcohol exposure (Anton and Becker 1995).

When analyzed together, both animal and clinical studies have provided corroborating evidence for the kindling hypothesis of AW. These findings indicate that in addition to the alcohol dose and the duration of alcohol exposure, a history of withdrawal episodes represents another critical factor determining the severity of a given withdrawal episode.

## Brain Mechanisms Underlying AW Kindling

Chronic alcohol exposure and subsequent withdrawal affect many neurochemical signaling systems in the CNS. Signal transmission among neurons occurs primarily through chemicals called neurotransmitters. These molecules are secreted by the signal-emitting cell and bind to docking molecules (i.e., receptors) on the signal-receiving cell. The interaction between a neurotransmitter and its receptor initiates a cascade of biochemical events in the signal-receiving cell. As a result, the signal-receiving cell may become easier or more difficult to excite, depending on the neurotransmitter involved. Neurotransmitters that increase the excitability of the signal-receiving cell are called excitatory neurotransmitters. Conversely, neurotransmitters that reduce the excitability of the signal-receiving cell are called inhibitory neurotransmitters. Alcohol generally has a suppressive effect on the CNS: It reduces the activity of excitatory neurotransmitters and their receptors and enhances the activity of inhibitory neurotransmitters and their receptors.

**Figure 1:**
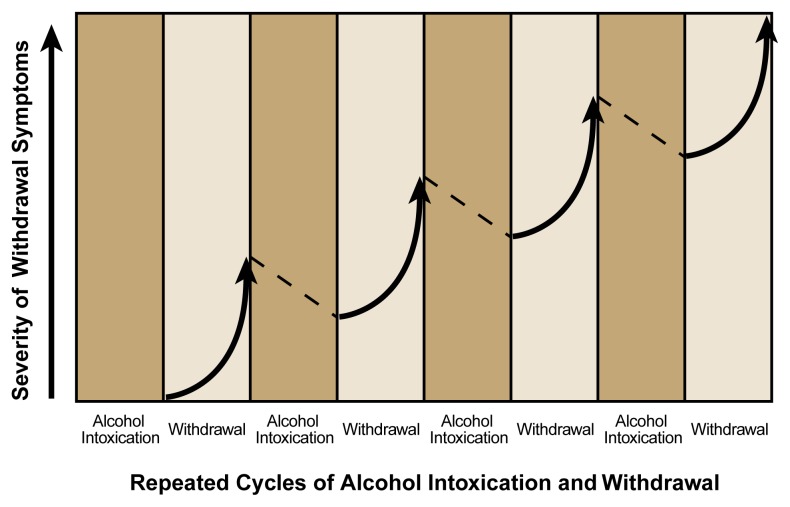
Graphic representation of the kindling concept during alcohol withdrawal. The term “kindling” refers to the phenomenon that people undergoing repeated cycles of intoxication followed by abstinence and withdrawal will experience increasingly severe withdrawal symptoms with each successive cycle.

After long-term alcohol exposure, the body activates a complex set of mechanisms to counteract the effects of alcohol’s persistent presence in the brain. These mechanisms promote the activity of excitatory neurotransmitter systems and suppress the activity of inhibitory neurotransmitter systems, thereby attempting to return brain function to a “normal” state in the presence of alcohol. When the individual stops drinking, however, these adaptive changes result in an imbalance in inhibitory and excitatory neurotransmission, resulting in CNS hyperexcitability that manifests itself as withdrawal symptoms. With a drinking pattern of repeated bingeing and abstaining, the imbalance occurring during withdrawal may accrue and intensify with each successive episode and may culminate in a state of persistent CNS hyperexcitability seen as a kindled (i.e., augmented) withdrawal response (see figure 1). The following sections review changes in inhibitory and excitatory neurotransmitter systems that play a role in mediating CNS activity and function during AW.

### Changes in Inhibitory Neurotransmission

**Figure 2:**
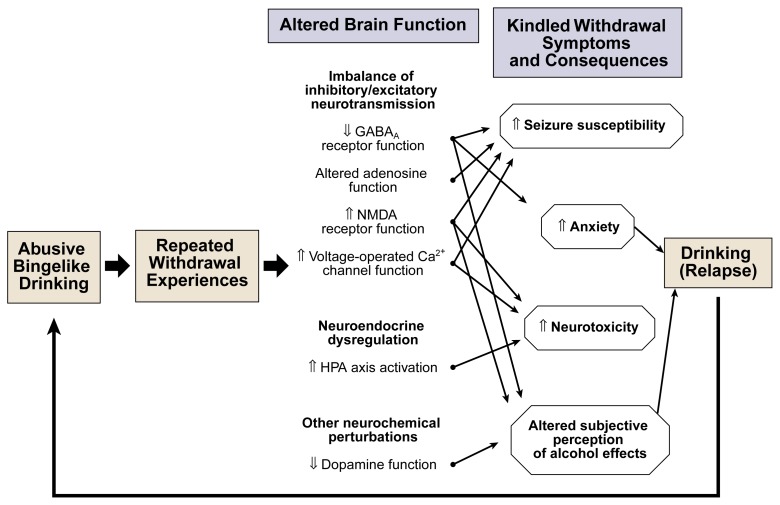
Possible mechanisms contributing to kindling during alcohol withdrawal. Repeated binge drinking followed by abstinence leads to repeated withdrawal episodes, resulting in increasingly severe alterations of brain functions. The alterations include (1) a progressive imbalance between suppressive (i.e., inhibitory) and stimulating (i.e., excitatory) influences (i.e., neurotransmission) on brain function, (2) disturbances in certain hormonal systems (i.e., neuroendocrine dysregulation), and (3) other neurochemical perturbations. The changes result in increasingly severe withdrawal symptoms, including seizures, anxiety, toxic effects on nerve cells (i.e., neurotoxicity), and altered perception of alcohol’s effects. Any of those symptoms may increase the patient’s potential for relapse and vulnerability to brain damage. GABA_A_ receptor: A receptor for the inhibitory neurotransmitter gamma-aminobutyric acid (GABA). NMDA receptor: A receptor for the excitatory neurotransmitter glutamate. HPA axis: A hormone system involving hormones produced in the hypothalamus, pituitary gland, and adrenal gland. Voltage-operated Ca^2+^ channel function: These calcium channels are located on the outer surface of the nerve cell. Electrical activity causes the channels to open, admitting calcium. Calcium entering the cell regulates many important processes related to cellular communication. ⇑ indicates an increase and ⇓ indicates a decrease in the brain function or withdrawal symptom listed. to increased seizure susceptibility upon cessation of alcohol exposure.

The primary inhibitory neurotransmitter in the mammalian brain is gamma-aminobutyric acid (GABA), which exerts its effects primarily through the GABA_A_ receptor (for more information on the mechanism of action of the GABA_A_ receptor, see figure 2). Interference with the actions of GABA and the GABA_A_ receptor—for example, treatment with agents that block the GABA_A_ receptor (i.e., receptor antagonists)—can cause convulsions, which are indicators of CNS hyperexcitability. Alcohol exposure results in activation of the GABA_A_ receptor, which in turn leads to reduced CNS excitability and may thus contribute to alcohol’s sedative effects (for a review, see Mihic and Harris 1997). In response to chronic alcohol exposure, the CNS adapts to the alcohol-induced GABA_A_ activation by reducing GABA-mediated neurotransmission.

Several studies performed in intact animals, isolated brain tissue slices, and cultured neurons have indicated that the effects of chronic alcohol exposure on GABA_A_ receptors may contribute to exacerbated withdrawal responses following multiple withdrawal episodes, as follows:

- Various studies using animals exposed to alcohol have demonstrated that animals with a history of multiple withdrawal episodes exhibited enhanced sensitivity to the convulsant properties of GABA_A_ receptor antagonists (Becker and Littleton 1996; Kokka et al. 1993; McCown and Breese 1993).
- After activation of GABA_A_ receptors, chloride ions enter the neuron; the resulting increase in the chloride concentration within the cell dampens neural activity. This flow of chloride ions into the cell was decreased in brain slices from the hippocampus^1^ of rats exposed to chronic intermittent alcohol treatment (Kang et al. 1996).
- Treatment of cultured mammalian cortical neurons with repeated cycles of alcohol exposure and withdrawal enhanced the cells’ sensitivity to treatment with GABA_A_ receptor antagonists that prevented the GABA-mediated flow of chloride ions into the cells (Hu and Ticku 1997).
- Rats with a history of multiple withdrawal episodes exhibited reduced GABA-mediated neural inhibition. Moreover, the subunit composition of the GABA_A_ receptor—each receptor consists of five protein molecules, or subunits—was altered in the hippocampus of these animals (Mahmoudi et al. 1997). This change in the structure of the GABA_A_ receptor may contribute to the reduced inhibitory actions of GABA and, consequently,

In addition to GABA, changes in the inhibitory actions of another neurotransmitter, adenosine, also have been implicated in kindling during AW (Jarvis and Becker 1998).

### Changes in Excitatory Neurotransmission

Along with decreased inhibitory neurotransmission, enhanced excitatory neurotransmission may contribute to the exacerbated withdrawal responses resulting from repeated withdrawal experiences. The primary excitatory neurotransmitter in the brain is glutamate, which can interact with several receptors. One of these receptors also can be activated by the substance *N*-methyl-d-aspartate (NMDA) and is therefore called the NMDA receptor. Excessive activation of this receptor induces seizures. (For more information on the function of this receptor, see figure 2.) The involvement of the glutamate-NMDA receptor system in kindling has been suggested by findings that animals with a history of multiple AW episodes exhibited increased sensitivity to NMDA-induced seizures compared with animals tested following a single withdrawal episode (Becker et al. in press). In cortical neurons grown in culture, repeated alcohol exposure and withdrawal increased the number of NMDA receptors on the cells. This increase was accompanied by a rise in the NMDA-induced flow of calcium ions through the receptor channel into the neurons, resulting in enhanced neural excitability (Hu and Ticku 1997).

Prolonged stimulation of NMDA receptors and calcium entry into brain cells can result in excessive cellular activity and, ultimately, neuronal death (i.e., excitotoxicity). In experiments using cultured tissue slices from rat hippocampus, NMDA-induced excitotoxicity was enhanced following repeated alcohol exposure and withdrawal (Becker and Littleton 1996), suggesting that this mechanism also may contribute to kindling during AW.

Another type of glutamate receptor is the kainate receptor, which also can be activated by the chemical kainate, but not by NMDA. Kainate receptors generally are located on different neurons than NMDA receptors. In contrast to the effects observed with NMDA, multiple withdrawal experiences in mice did not increase the animals’ sensitivity to the convulsant properties of kainate (Becker et al. in press). This observation suggests that the kindling process does not involve all glutamate receptors but is associated—at least initially—with selective neurochemical changes in specific brain regions. Recent electrophysiological data have provided additional support for this notion (Veatch and Gonzalez 1996).

In addition to the NMDA and kainate receptors, other excitatory neurochemical systems are likely responsive to repeated episodes of alcohol intoxication followed by withdrawal and, therefore, contribute to the kindling phenomenon. For example, repeated alcohol exposure and withdrawal may result in the perturbation of calcium channels that are activated not by neurotransmitter binding but by changes in the voltage difference (i.e., potential) between the inside and the outside of the neuron. The abnormal activation of such calcium channels also may alter calcium levels within the neurons and subsequently lead to neural damage. Further elucidation of brain mechanisms underlying kindling during AW should yield valuable insight into these processes and provide critically important guidance for the development and evaluation of new pharmacotherapies for managing AW.

## Treatment Implications of AW Kindling

Clinicians have used numerous strategies to manage AW. The primary goals of treating alcoholics during withdrawal are to prevent the occurrence of seizures and delirium tremens and to ameliorate discomfort related to autonomic instability (e.g., increased heart rate or blood pressure and sweating) and psychological instability (e.g., anxiety). Given the high rate of recidivism among alcoholics, many patients presenting for AW treatment probably have a history of detoxification. Other patients will likely experience additional withdrawal episodes in the future and are therefore at risk for more severe withdrawal symptoms during those future episodes as a result of kindling. Accordingly, the kindling phenomenon and the patient’s withdrawal history likely have a clinically significant effect on treatment strategies.

Some debate still exists among clinicians and researchers over whether all patients experiencing AW should be treated aggressively. On the one hand, many clinicians are concerned about the safety of available pharmacological treatments (e.g., sedatives, such as benzodiazepines) because of their abuse and dependence potential. This concern is particularly important, because the primary goal of alcoholism treatment is for the patient to become dependence free. Consequently, some clinicians may fear that they are substituting one dependence for another when using benzodiazepines for treating withdrawal. On the other hand, the growing body of evidence for withdrawal sensitization suggests that if left untreated, repeated withdrawal episodes (even if they are relatively mild) may progress to a more severe, life-threatening syndrome in the future.

Some experimental evidence has suggested that treatment of early withdrawal episodes may delay the development of later kindled withdrawal seizures (Ulrichsen et al. 1992; 1995). Both experimental and clinical studies have demonstrated, however, that repeated administration of sedative hypnotics for the treatment of multiple withdrawal episodes may be associated with serious drawbacks (Becker and Littleton 1996; Mayo-Smith and Bernard 1995). In particular, enhanced withdrawal seizures can occur as a result of withdrawal from the sedative hypnotics. Consequently, the development of safer and more effective treatments that could be used repeatedly for detoxification would be of great benefit. This area of research clearly warrants additional investigation.

## Clinical Significance and Implications of AW Kindling

### Association of Kindling and Relapse

Most animal models and clinical studies of kindling have focused on withdrawal-related seizures. Kindling during AW may have broader significance and clinical implications, however, if other withdrawal symptoms also could be exacerbated by repeated withdrawal experiences. For example, a progressive intensification of psychological symptoms of withdrawal (e.g., dysphoria^2^), as well as changes in the subjective perception of alcohol’s intoxicating effects, could substantially increase the patient’s motivation to resume drinking.

Several studies have suggested that changes in the brain mechanisms involved in mediating alcohol’s rewarding effects may become more pronounced following successive intoxication and withdrawal experiences. For example, when alcohol-dependent rats undergoing withdrawal were given the opportunity to self-administer alcohol, their alcohol consumption increased and became progressively more stable over successive withdrawal episodes (Roberts et al. 1996). Treatment with agents that affected the GABA system modified this withdrawal-related alcohol consumption. In other experiments, alcohol consumption in dependent rats undergoing withdrawal restored deficits in the brain levels of the neurotransmitter dopamine (Weiss et al. 1996). Dopamine-mediated neurotransmission in various brain regions has been shown to play a key role in mediating both alcohol’s rewarding effects and the dysphoria associated with withdrawal from chronic alcohol exposure (e.g., Koob et al. 1998).

Repeated withdrawal episodes, particularly if they occur in similar settings, also may contribute to relapse through a mechanism involving a conditioned withdrawal response. During a conditioned withdrawal response, environmental stimuli that are repeatedly associated with withdrawal symptoms (e.g., a physician’s office or hospital) may themselves become cues that trigger the neurochemical changes resulting in the physical and psychological withdrawal symptoms. Conditioned withdrawal-related responses, which reflect a kindlinglike process, may represent the biological basis for cue-induced alcohol craving in these circumstances. Further experimental exploration of these issues should provide valuable information regarding the possible influence of a patient’s withdrawal history on his or her risk for relapse. This knowledge is critical for developing effective treatment strategies for alcoholics who experience recurrent withdrawal episodes.

### Role of Kindling in Alcohol-Related Brain Damage and Cognitive Impairment

Multiple AW experiences also may render a person more vulnerable to brain damage. One potential mechanism contributing to this type of brain damage is the enhanced excitotoxicity associated with progressive increases in excitatory neurotransmission. In addition, withdrawal-induced persistent changes in certain hormonal systems affecting the CNS (i.e., neuroendocrine changes) may contribute to alcohol-related neuropathology.

One hormone system that may play a role in these processes is called the hypothalamic-pituitary-adrenocortical (HPA) axis, because the hormones involved in this system are secreted by a brain region called the hypothalamus, the pituitary gland, and the outer layer (i.e., cortex) of the adrenal gland. This hormone system is involved primarily in the body’s response to stress. AW activates the HPA axis, and the levels of hormones secreted by the adrenal cortex (i.e., corticosteroids) increase. Research indicates that this withdrawal-induced increase in corticosteroid levels is enhanced and more sustained in animals with a history of multiple withdrawal episodes (Becker and Littleton 1996). One group of corticosteroids, the glucocorticoids, influence neural excitability by interacting with certain receptors in the brain. Prolonged stimulation of these receptors as a result of elevated glucocorticoid levels in the blood may not only alter seizure susceptibility but also may produce neural damage, particularly in brain structures such as the hippocampus.

The progressive magnification of neurochemical and neuroendocrine alterations that occurs with successive AW experiences may result in enhanced vulnerability to alcohol-induced neurotoxicity. This neuronal damage, in turn, may underlie the cognitive deficits related to chronic binge-drinking patterns (Hunt 1993). In fact, both clinical and experimental findings have indicated that repeated AW is associated with increased cognitive dysfunction (Becker and Littleton 1996). This area of research also deserves greater experimental attention.

## Summary

Over the past two decades, a large body of clinical data has suggested that repeated AW may exacerbate the severity of future withdrawal episodes. Researchers have used a variety of animal models to further extend the clinical observations and to elucidate the mechanisms underlying this phenomenon (see figure 2). The results of these investigations have advanced the notion that the progressive intensification of withdrawal symptoms may be the manifestation of a kindling mechanism. Moreover, the findings indicate that this sensitization process reflects a persistent perturbation in excitatory and inhibitory influences on overall brain function. Kindling also may contribute to an exacerbation of the psychological components of withdrawal, which, in turn, may affect relapse risk. Finally, incremental changes in neurochemical and neuroendocrine systems following repeated withdrawal episodes may render the brain more vulnerable to neurological damage and associated cognitive impairments.

The potential consequences of kindling during AW highlight the clinical significance of the phenomenon and underscore the importance of considering this issue when making decisions about detoxification treatment. Given the high rate of recidivism among alcoholics, each withdrawal episode may perhaps best be viewed not as an isolated event but as part of a potentially long-term process that can lead to dangerous exacerbation of withdrawal symptoms with each subsequent episode. Continued research in this area will provide insight and appropriate guidance for the development of new and more effective treatment strategies for alcohol detoxification as well as for the long-term management of alcohol abuse and dependence.

## Footnotes

1 The hippocampus is a brain region involved in memory and learning; its function is affected by alcohol.

2 Dysphoria is a state characterized by depression, anxiety, and emotional discomfort and involves the inability to derive pleasure from normally rewarding events.
